# Interorganizational Relationships Within State Tobacco Control Networks: A Social Network Analysis

**Published:** 2004-09-15

**Authors:** Nancy Mueller, Melissa Krauss, Douglas Luke

**Affiliations:** Center for Tobacco Policy Research, Saint Louis University School of Public Health; Center for Tobacco Policy Research, Saint Louis University School of Public Health, St. Louis, Mo; Center for Tobacco Policy Research, Saint Louis University School of Public Health, St. Louis, Mo

## Abstract

**Introduction:**

State tobacco control programs are implemented by networks of public and private agencies with a common goal to reduce tobacco use. The degree of a program's comprehensiveness depends on the scope of its activities and the variety of agencies involved in the network. Structural aspects of these networks could help describe the process of implementing a state's tobacco control program, but have not yet been examined.

**Methods:**

Social network analysis was used to examine the structure of five state tobacco control networks. Semi-structured interviews with key agencies collected quantitative and qualitative data on frequency of contact among network partners, money flow, relationship productivity, level of network effectiveness, and methods for improvement.

**Results:**

Most states had hierarchical communication structures in which partner agencies had frequent contact with one or two central agencies. Lead agencies had the highest control over network communication. Networks with denser communication structures had denser productivity structures. Lead agencies had the highest financial influence within the networks, while statewide coalitions were financially influenced by others. Lead agencies had highly productive relationships with others, while agencies with narrow roles had fewer productive relationships. Statewide coalitions that received Robert Wood Johnson Foundation funding had more highly productive relationships than coalitions that did not receive the funding.

**Conclusion:**

Results suggest that frequent communication among network partners is related to more highly productive relationships. Results also highlight the importance of lead agencies and statewide coalitions in implementing a comprehensive state tobacco control program. Network analysis could be useful in developing process indicators for state tobacco control programs.

## Introduction

Tobacco control activities in the United States predominately occur in highly complex, comprehensive state tobacco control programs. These programs are usually considered comprehensive based on the scope of activities implemented to reduce tobacco use. For example, as outlined in the Centers for Disease Control and Prevention's (CDC's) *Best Practices for Comprehensive Tobacco Control Programs — August 1999*, a comprehensive program should include some level of activity in community programs, chronic disease programs, school programs, enforcement, statewide programs, counter-marketing, cessation programs, surveillance and evaluation, and administration and management (1).

Many activities are said to be included in a comprehensive program, which suggests many organizations must be involved. Therefore, a state tobacco control program's comprehensiveness refers not only to its activities but also to its multifaceted structure. Tobacco control programs have complicated and ambitious goals that cannot be achieved solely by one agency, but through the efforts of many agencies. While usually led by a state department of health or an independent tobacco control agency, a state's efforts involve a wide range of other stakeholders, such as contractors with the lead agency, regional and statewide coalitions, and voluntary agencies. These agencies attempt to work in partnership toward their common goal of reducing tobacco use in their state through strategic planning, policy implementation, prevention and cessation activities, and advocacy. Collaboration and coordination are essential parts of this process. Collaboration between public health groups and private organizations has been argued to be an effective tool for advancing a variety of tobacco control initiatives (2). In fact, the creation of public and private partnerships and the development of a shared leadership model were cited as some of the key contributing factors to providing the foundation for a coordinated approach in the evolution of the Massachusetts Tobacco Control Program (3).

Typically, evaluations of state programs have concentrated on the effectiveness of tobacco control activities in decreasing tobacco use (4-10). State programs have also been evaluated on program inputs, such as the level of funding the state has dedicated to its tobacco control program (11-13), the strength of the state's tobacco control policies (14,15), or, to a lesser extent, political and financial climates and measures of organizational capacity (16). Because collaboration is an important component of a state's program, the relationships among the agencies within the network should also be evaluated to help describe the process of implementing a coordinated and comprehensive tobacco control program. A well-connected tobacco control network could improve a state's tobacco control effort through more efficient and effective use of knowledge and resources. However, to our knowledge, the interorganizational structures and relationships of state tobacco control networks have not yet been examined.

Social network analysis is a particularly useful quantitative analytic method that can be used to examine relationships among social entities, such as the various agencies involved in a state's tobacco control program. This type of analysis can be used to address such questions as how hierarchical a communication structure is, or which entities have more control over information or resources than others in the network. Social network analysis has been used in a wide range of social and behavioral science disciplines, including tobacco behavior research, to study the influences of peer group social structure on youth smoking (17-21). This technique has also been used to examine the structure of interorganizational relations and how that structure can influence organizational behavior (22). Social network analysis has helped explain the relationship between how central an organization is within a network and how powerful it is. It also explains the relationship between how the structure of interorganizational relations influences an organization's strategies and political behavior and how the organization secures resources (22). Social network analysis has been used in a wide variety of areas to describe interorganizational relationships, including those addressing health and policy. For example, social network analysis was used to investigate the structure of the Canadian women's movement by studying relationships among national women's groups; to study relationships among stakeholder organizations in the mental health policy area; to examine interorganizational relations among HIV/AIDS service agencies; and to analyze the network of services for pregnant low-income women (23-26).

The purpose of this study is to examine the interorganizational relationships of state tobacco control networks using social network analysis. Our study adds to the literature on the evaluation of state tobacco control programs by leading to a greater understanding of the intricacies and complexities of tobacco control networks. Specific objectives of the study are to 1) examine relationships among tobacco control agencies within state programs based on their communication, productivity, and exchange of funding, 2) identify the most important actors within the tobacco control networks and describe how they relate to other actors, and 3) investigate the structure of tobacco control networks by comparing and contrasting five state network structures.

## Methods

### Project overview

The Center for Tobacco Policy Research is conducting a multiyear process evaluation on the status of 10 state tobacco control programs. A cross-sectional study design was used to evaluate the process of organizing and conducting statewide tobacco prevention activities. One of the specific objectives of the study was to examine the interorganizational relationships of the tobacco control network.

### Sample

To obtain a diverse sample of states, states were selected based on 1) geographic location; 2) level of program capacity (e.g., funding level, age of program)To obtain a diverse sample of states, states were selected based on 1) geographic location; 2) level of program capacity (e.g., funding level, age of program); 3) presence of tobacco farming; and 4) type of lead agency (state health departments or independent organization). The 10 selected states thus represented a variety of tobacco control programs from across the country. This paper presents the network analysis results from the first set of states evaluated: Washington (evaluated June 2002), Indiana (July 2002), Wyoming (October 2002), New York (December 2002), and Michigan (February 2003).

A modified fixed-list sampling method was implemented to identify the key partner agencies of each state tobacco control program (27). The tobacco control manager from the lead agency was first contacted and asked to compile a list of partners that contributed substantially to the program or had a unique role. The research team and the tobacco control manager then discussed the list to finalize the number of agencies and individuals who would be invited to participate in an interview. The statewide coalition director verified that all major agencies were represented. During the interviews, each agency was asked questions about the other agencies in the network. Individuals interviewed also had an opportunity to suggest additional participants. The average number of agencies interviewed within each state was 14.

### Data collection

For each agency in the tobacco control program, a key informant was identified and asked to participate in an interview. This key informant was the staff member most familiar with the agency's tobacco control activities. The semi-structured in-depth interview collected quantitative and qualitative data on network characteristics, political support for tobacco control, the financial climate of the state, use of the CDC's *Best Practices for Comprehensive Tobacco Control Programs*, and organizational capacity. Trained interview teams conducted the interviews either inperson or over the telephone. Approximately 54% of the interviews were conducted in person. The average interview lasted 73 minutes.

### Quantitative network measures

Throughout the interviews, we collected data about each agency's interaction with other agencies within the network. The quantitative relational constructs measured were the frequency of contact (through meetings, phone calls, or e-mails) among agencies, the flow of money among agencies, and the perceived productivity of agency relationships. Appendix A shows an example of the network data collection form for one state. Each participant was asked questions about each partner agency in the state's tobacco control network. We followed typical social network analysis procedures to prepare data for analysis (28). Missing values were indicated when participants felt they were unable to answer the question because of limited contact or knowledge.

For contact frequency, if multiple respondents were interviewed from one agency (which usually only occurred with the lead agency), we averaged respondent values to produce one final value. When one response was missing for a pair of agencies, we used the response given by the other agency. Although not ideal, this type of imputation of missing network data is common. Basic network measures such as betweenness have been shown to be reliable with as much as 25% missing data (29). Answers were averaged from each pair of agencies to determine one level of contact. This is important because although individual reports of contact level may vary, it was assumed that interagency contact was symmetric. That is, if Agency A had a meeting with Agency B, this naturally means that Agency B met with Agency A. We then dichotomized the average score into "at least monthly contact" and "less than monthly contact."

For money flow, if multiple respondents were interviewed from one agency, we used the responses given by the most senior staff member interviewed. When disagreement arose on the perception of money exchange between a pair of agencies, we contacted each agency to determine the correct response. If one response was missing for a pair of agencies, we used the response given by the other agency in the pair. The money construct was dichotomized into "send money" or "do not send money."

For relationship productivity, if multiple respondents were interviewed from one agency, we averaged respondent values to produce one final value. When one response was missing for a pair of agencies, we used the response given by the other agency. When both responses were missing, we chose "neutral" as the response. This decision was made so that we would not lose the entire node in the network, nor make any assumptions on the direction of the response, given no data from either partner. We dichotomized the scores into "very productive" relationships, the highest possible productivity response, and "not very productive" relationships, which includes "counter-productive," "very unproductive," "somewhat unproductive," "neutral," and "somewhat productive."

### Social network analysis

We performed the social network analysis using graphic and statistical methods. Graphs based on each of the three constructs described above (contact frequency, money flow, and relationship productivity) were created to visually depict the relationships in each network. Statistical analyses provided measures both at the agency and network levels. Graph construction and social network analyses were conducted using UCINET Social Network Analysis Software Version 6 (Analytic Technologies, Inc, Harvard, Mass) Pajek Program for Large Network Analysis (Vlado, Ljubljana, Slovenia), and NetDraw Network Visualization (Analytic Technologies, Inc, Harvard, Mass).

GlossaryFor the discussion in this article, these nontechnical network analysis definitions may be useful (28):

Connectedness: : A network graph is connected if there is a path or tie between every pair of actors in the graph. Therefore, all pairs of actors are reachable in a connected graph.
Density: is defined here as the proportion of possible lines or ties that are actually present in a network graph. Because it represents a proportion, density ranges from 0 to 1.
Centrality: provides a measure of how central an actor is within a network. Actors that are highly central are interpreted as controlling the flow of information or resources within the network.
Betweenness: is a measure of centrality based on how often an actor in a network is found in the shortest pathway between other actors in the network. The equation for normalized betweenness can be found in Appendix B. It is used in this study to evaluate frequency of contact and control over network communication. Higher scores indicate higher control.
Prestige: is a value that is commonly examined in a directional relationship as a way to identify prominent actors in a network. A prestigious or prominent actor is one that is the object or recipient of many ties in the network.
Normalized indegree: is used to measure prestige; it indicates the number of directional ties terminating at or pointing toward an actor. A higher indegree score indicates higher prestige.

#### Contact frequency

One important use of social network analysis is to identify the most important actors in a network, which are considered to be in strategic locations within the network (28). Our measure of contact frequency is nondirectional, meaning that the relation between two actors in the network holds simultaneously for both actors. So, if Agency A has contact with Agency B at least monthly, then Agency B must also have contact with Agency A. One way to determine the important actors with this type of relation is to examine centrality using the measure of betweenness. We examined the normalized betweenness of each actor (30).

#### Flow of money

Exchange of money among network partners was a directional relation, meaning that the tie between two actors has an origin and a destination. In this case, Agency A may send money to Agency B, but not necessarily vice versa. For this construct, we used Taylor's influence to measure the amount of financial influence of one agency over another (31). (Appendix B provides more information on Taylor's influence.) A negative influence value indicates that an agency had a preponderance of receiving over sending money to other agencies in the network, a positive value indicates a preponderance of sending over receiving, and a neutral value indicates a balance of sending and receiving.

#### Relationship productivity

Our productivity variable was also a directional relation. Agency A may feel they have a productive relationship with Agency B, but the feeling may not be mutual. A common way of identifying prominent actors in a directional relation is by examining prestige, which we measured using normalized indegree (28). (See Appendix B for definition and equation.) In our study, a prominent or prestigious agency would be one with whom many others felt they had highly productive relationships. A higher indegree score indicates higher prestige.

#### Group-level indices

In addition to the actor-level measures described above, we also examined some group-level indices to facilitate comparisons across networks and states. A group betweenness centralization index was calculated for each contact frequency network, which indicates variability of the betweenness of members of the network. A high betweenness centralization score indicates a hierarchical network structure, where it is more likely that a single agency in the network is quite central, while remaining agencies are less central. We did not examine a group-level index for prestige because little research has been done to adequately develop and validate network-level prestige indices (28). We also examined the density of each network for contact frequency, relationship productivity, and money flow. Density indicates the proportion of all possible ties that are actually present in the network. The above constructs were also compared with other state network variables, such as funding level, receipt of the Robert Wood Johnson Foundation (RWJF) Smokeless States grants, and location of partner agencies.

### Qualitative network measures and analysis

Qualitative network constructs were collected via open-ended questions about the perceived effectiveness of the state's tobacco control network and suggestions for methods to improve the network's effectiveness. Each interview was transcribed verbatim and imported into the qualitative data management software NUD*IST (QSR International Pty Ltd, Melbourne, Australia). Each transcript was then coded using a detailed codebook developed during the pilot test by two trained staff members. Inter-rater reliability for coding was 83.7%. The coded text units were entered into NUD*IST, and a report was generated for each construct (e.g., network, financial climate). Analysis teams consisting of two trained staff members independently analyzed the reports to identify major themes or ideas. The team then met to discuss the results and arrive at consensus on major themes.

## Results

### Basic network characteristics

The five state tobacco control programs represented a variety of funding levels, network sizes, and geographic locations (Table 1). Annual funding levels for entire state programs ranged from $4.2 million (Wyoming, fiscal year 2003) to $52.3 million (New York, fiscal year 2003). According to the CDC's minimum recommendations for state tobacco control funding (1), Michigan had low funding (10% of CDC's recommendation), New York, Wyoming and Washington had middle funding levels (55%, 57%, and 62%), and Indiana had a high funding level (97%). Three of the statewide coalitions received a Smokeless States grant from the RWJF at the time of the evaluation. The average number of partner agencies per state network was 14, and the number of cities where partner agencies were located ranged from a few (two in Indiana) to as many as 11 (New York).

Four of the tobacco control networks were led by the tobacco control program within the state's department of health. The exception was Indiana's program, which was led by an independent tobacco control agency. Lead agencies usually included as key partners in the network were voluntary agencies, advocacy agencies, statewide and regional coalitions, and contractors with the lead agency. Partner agencies unique to some states included government agencies, political figures, trust fund agencies, media firms, and funding agencies. (Appendix C lists agencies, along with their abbreviations, included in each state network.)

### Monthly contact networks

Figure 1 depicts the monthly contact network for each of the five state programs. The amount of control over communication flow each agency had relative to other agencies in the network is based on centrality betweenness scores presented in Table 2 (the higher the score, the more control over communication flow in the network).

Figure 1Monthly contact network for five state tobacco control programs. A line connects two agencies that had contact with each other *at least* once a month via meetings, phone calls, or e-mails. Colored dots represent the amount of control over communication flow each agency had relative to other agencies in the network as determined by scores for betweenness. All acronyms are spelled out in Appendix C.Flow chart

**Figure:**
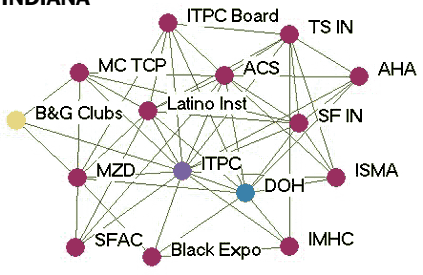
INDIANA

**Figure:**
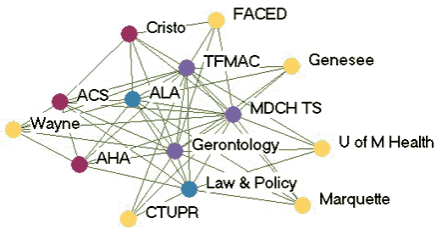
MICHIGAN

**Figure:**
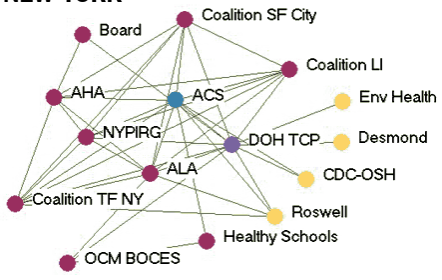
NEW YORK

**Figure:**
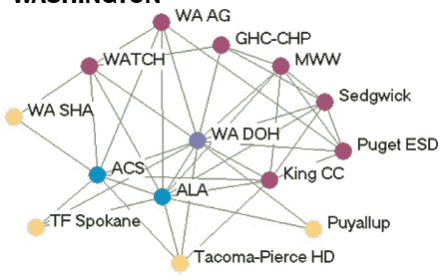
WASHINGTON

**Figure:**
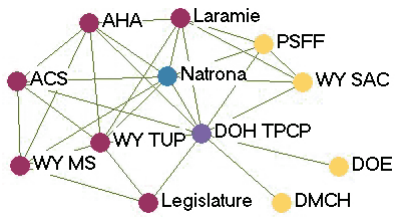
WYOMING

**Figure:**
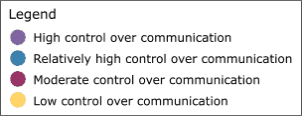
Legend

**Table:** Five state tobacco control networks – monthly contact

**State**	**High control over communication**	**Relatively high control over communication**	**Moderate control over communication**	**Low control over communication**
**Washington**	WADOH	ACS	WATCH	WASHA
		ALA	WAAG	TFSpokane
			GHC-CHP	Puyallup
			MWW	Tacoma-PierceHD
			Sedgewick	
			PugetESD	
			KingCC	
**Indiana**	ITPC	DOH	MC TCP	B&G Clubs
			ITPC Board	
			TS IN	
			AHA	
			ACS	
			Latino Inst	
			SF IN	
			MZD	
			ISMA	
			SFAC	
			Black Expo	
			IMHC	
**Wyoming**	DOH TPCP	Natrona	ACS	PSFF
			AHA	WY SAC
			Laramie	DOE
			WY MS	DMCH
			WY TUP	
			Legislature	
**New York**	DOH TCP	ACS	Board	Env Health
			Coalition SF City	Desmond
			AHA	CDC-OSH
			NYPIRG	Roswell
			ALA	
			Coalition TF NY	
			OCM BOCES	
			Healthy Schools	
**Michigan**	TFMAC	Law & Policy	Cristo	Wayne
	MDCH TS	ALA	ACS	FACED
	Gerontology		AHA	Genesee
				U of M Health
				Marquette
				CTUPR

Two of the states presented contrasting patterns of network communication structure: New York and Michigan. New York is an example of a very hierarchical structure because of its high centralization index (40.8%), high mean betweenness score (5.1), and standard deviation (11.0). Most agencies in the New York network had frequent contact with one central agency (the lead agency New York State Department of Health Tobacco Control Program), which also had the most control over communication flow within the network, indicated by its high betweenness score (43.1). Betweenness scores dropped substantially for other partner agencies. The density of New York's network was the lowest of the five states (0.39). Figure 1 shows that many agencies had very little contact with other agencies. An agency would likely have to go through the lead agency to communicate with another agency. From qualitative analysis, we found that network partners believed the network was improving and had potential, but thought it still needed to improve its coordination and communication.

Michigan, on the other hand, had a much flatter, or nonhierarchical, communication structure. This structure is reflected by its low centralization index (10.4%), and the low mean betweenness score (3.5) and standard deviation (5.2). Partner agencies had frequent contact with many other agencies in the network. Three agencies, including the lead agency Michigan Department of Community Health Tobacco Section, the statewide coalition Tobacco Free Michigan Action Coalition (TFMAC), and the contractor Gerontology, had higher control over communication flow than other agencies in the network. Although these three agencies had higher relative betweennness scores within their own network, their scores were not very high compared to agencies in other state networks. Michigan's density (0.58) was higher than that of the other four states. In contrast to New York's contact graph (Figure 1), Michigan's figure shows a much more connected communication network. There were multiple routes for information flow from agency to agency. Michigan's partner agencies felt their network was very effective and had improved over the last few years. They believed TFMAC and a Smokefree Regulations Task Force, a group of partners focusing on clean indoor air efforts, were important network components that facilitated the process of bringing stakeholders together. Partners felt this helped strengthen the network's ability to work more efficiently. Yet partners thought communication could be improved by openly sharing agencies' priorities and activities.

In examining all five states, a few patterns emerged. The lead agency usually had the highest control over communication flow, which intuitively makes sense. However, statewide coalitions, which are known to bring stakeholders together on tobacco issues, usually did not have the highest communication control, except in Michigan. Most network communication structures were relatively hierarchical, except Michigan, which had a much flatter structure. The average density of the contact networks was 0.47. The densities ranged from 0.39 in New York to 0.58 in Michigan.

### Money flow networks

Figure 2 shows the money flow network for each state. Table 3 presents the financial influence scores for each agency in each network. In most states, the lead agency had the highest financial influence over the network, which corresponds with their duties as fiscal oversight agencies. The exception was Wyoming, where the legislature was included as a partner in the network. Due to the legislature's role in appropriating funding, it had the highest financial influence. Statewide coalitions were usually highly influenced by others in the network, except in Indiana. The statewide coalition Tobacco Smart Indiana did not send or receive money from anyone in the network at the time of the study. At the time of the evaluation, the coalition was in transition because of a loss of funding.

Figure 2Money flow networks for five state tobacco control programs. Arrows indicate direction of money flow, and colored dots represent the relative amount of financial influence each agency had over the rest of the network. All acronyms are spelled out in Appendix C.Flow chart

**Figure:**
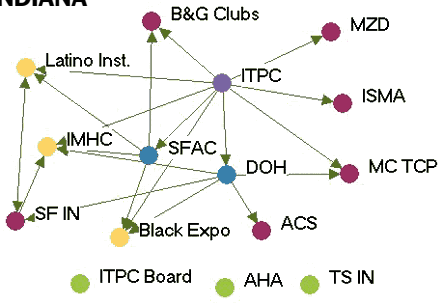
INDIANA

**Figure:**
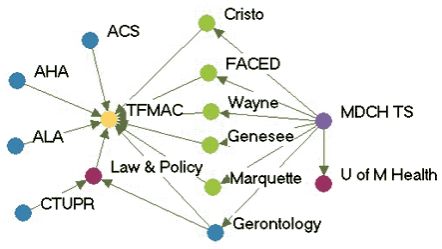
MICHIGAN

**Figure:**
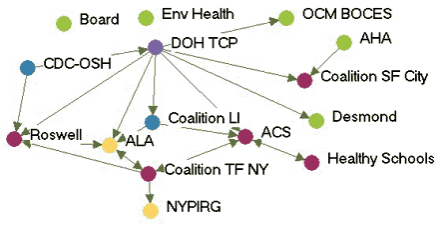
NEW YORK

**Figure:**
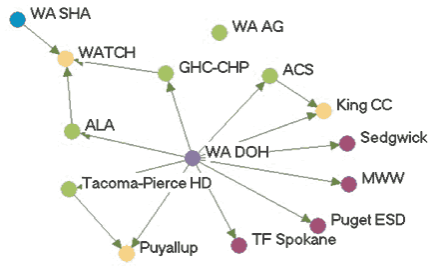
WASHINGTON

**Figure:**
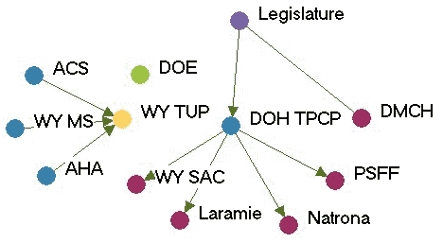
WYOMING

**Figure:**
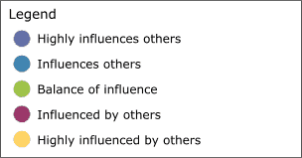
Legend

**Table:** Five state tobacco control networks – money flow

**State**	**Highly influences others**	**Influences others**	**Balance of influence**	**Influenced by others**	**Highly influenced by others**
**Washington**	WADOH	WASHA	ALA	Sedgewick	WATCH
			Tacoma-Pierce HD	MWW	Puyallup
			GHC-CHP	PugetESD	KingCC
			WAAG	TFSpokane	
			ACS		
**Indiana**	ITPC	SFAC	ITPC Board	SF IN	Latino Inst.
		DOH	AHA	B&G Clubs	IMHC
			TS IN	MZD	Black Expo
				ISMA	
				MC TCP	
				ACS	
**Wyoming**	Legislature	DOH TPCP	DOE	DMCH	WY TUP
		ACS		PSFF	
		WY MS		Natrona	
		AHA		Laramie	
				WY SAC	
**New York**	DOH TCP	Coalition LI	Board	Roswell	ALA
		CDC-OSH	Env Health	ACS	NYPIRG
			OCM BOCES	Coalition TFNY	
			AHA	Healthy Schools	
			Desmond	Coalition SF City	
**Michigan**	MDCH TS	Gerontology	Cristo	Law & Policy	TFMAC
		ACS	FACED	U of M Health	
		AHA	Wayne		
		ALA	Genesee		
		CTUPR	Marquette		
				

Many of the states with greater funding levels had more complex money flow networks, such as Indiana, New York, and Washington. In these states, money flowed not only from the lead agency to other agencies but also flowed among contractors and coalitions. Wyoming, which had a low funding level, had the simplest money flow network, with money mainly flowing from the legislature to the lead agency and then to contractors or coalitions. Some money was also given by other agencies to the statewide coalition. Network density related to money flow varied little among the states. The average density was 0.09.

### Highly productive relationships

Figure 3 depicts the productivity of relationships in the five state networks.  Table 4 presents the prestige score (normalized indegree) for each agency in each network.

Figure 3Productivity of relationships in the five state networks. An arrow from A to B indicates that Agency A felt it had a *very* productive relationship with Agency B. A bidirectional arrow indicates that both agencies agreed that their relationship was very productive. Colored dots represent the prestige each agency had relative to other agencies in the network. All acronyms are spelled out in Appendix C.Flow chart

**Figure:**
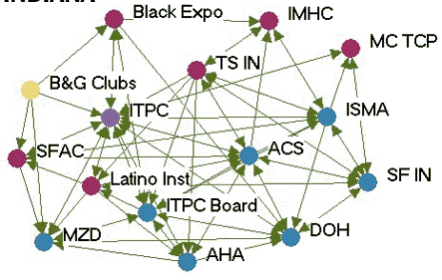
INDIANA

**Figure:**
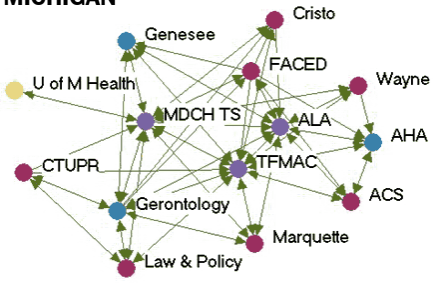
MICHIGAN

**Figure:**
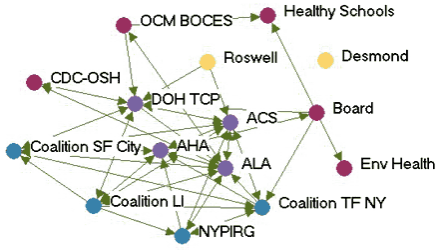
NEW YORK

**Figure:**
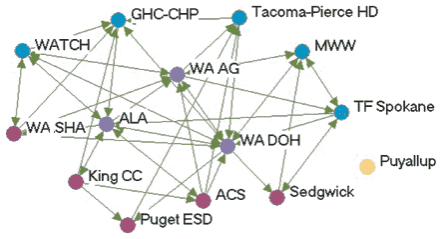
WASHINGTON

**Figure:**
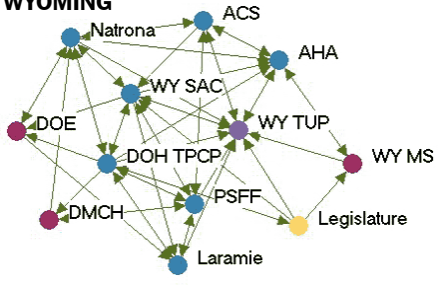
WYOMING

**Figure:**
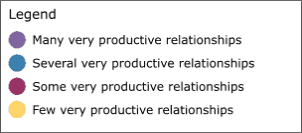
Legend

**Table:** Five state tobacco control networks — very productive relationships

**State**	**Many very productive relationships**	**Several very productive relationships**	**Some very productive relationships**	**Few very productive relationships**
**Washington**	WADOH	WATCH	WASHA	Puyallup
	WAAG	GHC-CHP	KingCC	
	ALA	Tacoma-Pierce HD	PugetESD	
		MWW	ACS	
		TFSpokane	Sedgewick	
**Indiana**	ITPC	MZD	Black Expo	B&G Clubs
		ITPC Board	SFAC	
		AHA	Latino Inst	
		ACS	TS IN	
		DOH	IMHC	
		ISMA	MC TCP	
		SF IN		
**Wyoming**	WYTUP	DOH TPCP	DOE	Legislature
		PSFF	WY MS	
		Laramie	DMCH	
		AHA		
		ACS		
		WY SAC		
		Natrona		
**New York**	DOH TCP	Coalition SF City	CDC-OSH	Roswell
	ACS	Coalition LI	OCM BOCES	Desmond
	AHA	Coalition TF NY	Healthy Schools	
	ALA	NYPIRG	Board	
			Env Health	
**Michigan**	MDCH TS	Genesee	Cristo	U of M Health
	ALA	AHA	FACED	
	TFMAC	Gerontology	Wayne	
			ACS	
			Marquette	
			Law & Policy	
			CTUPR	

In Washington, the lead agency, Washington State Department of Health Tobacco Prevention and Control Program (WA DOH), had the highest prestige (normalized indegree 69.2), followed by the American Lung Association-Washington State Branch and the Washington Office of the Attorney General (both normalized indegree 46.2). The WA DOH was highly regarded by partner agencies. Furthermore, partner agencies were pleased that the state secretary of health had made tobacco control her highest priority. The state attorney general had been one of the lead negotiators in the Master Settlement Agreement and was overwhelmingly identified as a tobacco control champion. The statewide coalition Washington Alliance for Tobacco Control and Children's Health (WATCH) ranked in the middle of other Washington agencies in prestige (normalized indegree 30.8). At the time of the evaluation, WATCH was going through a transition because of a recent loss of funding and was reevaluating its role in the tobacco control network. Many partners were uncertain of the coalition's existence or future plans. Washington's productive relationships network had a low density compared to the other states (0.27).

Wyoming's statewide coalition, Wyoming Tobacco Use Prevention (WY TUP), had the greatest prestige score in its state (normalized indegree 81.8). The lead agency, Wyoming Department of Health Substance Abuse Division, Tobacco Prevention and Control Program (DOH TPCP), on the other hand, had a much lower score (normalized indegree 45.5). Again, these results were supported qualitatively. Partners were very pleased with WY TUP and its accomplishments. However, they believed the DOH TPCP, which houses the tobacco control program, did not provide enough support for tobacco control. In Wyoming, the Legislature had the lowest prestige score (normalized indegree 9.1) due to sentiment that the legislature as a whole was unsupportive of tobacco control. Partners believed that only a few legislators made tobacco control a priority. The Department of Education and Department of Maternal and Child Health in Wyoming also received relatively low scores in prestige. Partners believed those agencies were not as engaged in tobacco control as they could be.

Although problems may be encountered with the lead agency in some states, it usually had a very high number of productive relationships (if not the highest) across states. Statewide coalitions funded by the RWJF (New York, Wyoming, and Michigan) had higher prestige scores than coalitions that did not receive RWJF funding and were in transition (Washington and Indiana). The average prestige score for RWJF-funded coalitions was 69.9, compared to 29.7 for non-RWJF–funded coalitions. Agencies with narrowly defined roles, such as contractors or agencies with a local focus, had fewer highly productive relationships, while state level agencies usually had higher numbers of productive relationships. The average density of the productivity networks was 0.34. The results suggested a relationship between contact network density and productivity network density. Michigan had the highest productivity density (0.41) and also had the highest contact density (0.58). New York (0.25) and Washington (0.27) had lower productivity densities and also had lower contact densities (New York, 0.39 and Washington, 0.44).

## Discussion

This paper presents a new construct that can be used to examine state tobacco programs: network structure. Using social network analysis to examine five state tobacco control programs led to some important observations. Key actors in the networks were highlighted, such as the lead agency of the programs. In all five states, lead agencies had high control over communication flow, many highly productive relationships, and much financial influence over the networks. The financial stability of statewide coalitions also influenced network structure. Statewide coalitions had many highly productive relationships in some states, but not as many in other states. This difference could be explained by the funding status of the coalitions. Those with RWJF funding scored higher in productivity than coalitions without RWJF funding. Funding is necessary for sustaining a stable statewide coalition and for building and maintaining high-quality relationships with others in the state.

Some patterns between network structure and basic descriptive network characteristics also emerged. For example, results suggested that geographic dispersion of a network could play a role in communication among agencies. Densities of contact networks appeared to be higher for states with partners in fewer locations and vice versa. New York had the greatest number of partner agency locations (11) and the lowest density (0.39). Conversely, Indiana had a low number of partner agency locations (2) and a high contact density (0.50). This does not suggest that tobacco control efforts should be concentrated in only a few areas of the state. In fact, it is important to have partners in many areas of the state to reach citizens with messages of prevention and cessation and to create policy change. This does suggest, however, that increased efforts may be needed to facilitate communication among agencies located throughout the state.

While the results of this descriptive study cannot directly assess causality, it was clear that communication among agencies was an important factor for having productive relationships. Results suggested that states with more dense contact networks had more dense productivity networks. Michigan had the highest contact density (0.58) and the highest productive relationships density (0.41). New York had the lowest density for contact (0.39) and productive relationships (0.25). This is logical because a productive relationship can only occur when at least some contact occurs between agencies. However, frequency of contact could be a factor here. The more often communication occurs among agencies, the more they work together and the more productive they feel their relationship is. It is relatively easy to investigate the amount of contact occurring among agencies. Little contact among agencies could be a symptom of other problems in the network that may lead to lower productivity.

To further pursue this, we calculated the relationship between contact and productivity for each of the five states by determining the graph correlation using the quadratic assignment procedure (32). Table 5 presents these correlations and shows that for these states there is a significant positive correlation between contact and relationship (average correlation = .56). Therefore, we see that tobacco control agencies that have more frequent contact with each other are more likely to report highly productive relationships.

Surprisingly, a relationship between funding level and network connectedness was not suggested by our results. Both New York and Indiana had high funding dollar amounts, but Indiana's network seemed very well connected, while New York's network was less connected. Michigan had a lower funding dollar amount and had a very connected network. Therefore, funding level does not seem to be a driving force for how connected a state program can be.

Nuances in state tobacco control programs also affected the structure of the networks. For example, Wyoming's tobacco control program had the only lead agency that was placed under the Substance Abuse Division at the Department of Health. Partners believed the program did not provide enough support for tobacco control, which caused this lead agency to have fewer productive relationships than lead agencies in the other states. The Washington Attorney General's prominent role in the Master Settlement Agreement and continued support for the program made the attorney general a unique partner in the network, and partners believed their relationships with the attorney general were highly productive.

Results of this study should be interpreted with some caution. Because our sample size included only five state networks, results are not very generalizable. Furthermore, state networks presented do not include all agencies involved in the state network. A limited number of agencies could be interviewed because of the very large number of agencies involved in a state's program and the study team's limited resources. However, the type of sampling methodology employed here, which included a list of key partners identified by tobacco control program managers and the addition of some other agencies as suggested by the key partners, resulted in including the most important tobacco control agencies in the evaluated tobacco control network. Although only one individual per agency was usually interviewed, we believe those responses represent the viewpoint of the entire agency on quantitative network constructs. When multiple individuals from an agency were interviewed, their responses for quantitative constructs were highly correlated. The productivity of relationships was dichotomized as "very productive" or "not very productive."  Dichotomization of this variable was necessary for analysis, and we chose to use "very productive" as the cutoff to highlight the highest productivity level between agencies. Doing so could be a disadvantage because of the loss of variability in the measure. Finally, our analysis is based on reports of individuals from partner agencies, which may not be accurate, or at times were simply missing. Reported responses may differ from actual, observed interactions among partners. Possible bias in reporting or from missing data is an inherent limitation in key informant interviews.

The CDC has developed an extensive logic model for tobacco prevention in an effort to identify specific outcome indicators for comprehensive tobacco control programs (33). The emphasis in the CDC logic model is on short-, medium-, and long-term outcomes, such as smoking initiation rates, cigarette prices, and existence of tobacco control policies.

The work presented in this paper represents a first step in developing measures of *process indicators* as opposed to outcome indicators. That is, very little is known about the organizational structures and processes that may influence successful tobacco control outcomes. Investigating structures of state tobacco control networks can help shed light on the highly complex process of coordinating a state tobacco control program. Social network analysis provides an objective and relatively simple and inexpensive way to examine this process. One important way this type of analysis could be used is to examine the effect of state budget cuts on state tobacco control programs. Other than tracking the amount of money states spend on tobacco control, it is difficult to measure the impact of budget cuts on the process of implementing a statewide program. Network structure could be compared before and after budget cuts to determine changes in network characteristics and size. More importantly, future research could compare network analysis measures with the success of state programs in decreasing smoking rates or passing tobacco control policies to establish the link between network characteristics as process and program outcome indicators. Furthermore, this type of organizational network analysis represents a new way to perform program evaluation, in which the emphasis is less on simple counts of program activities and more on the documentation of structural and process changes that result from effective programs (26). In summary, network analysis of state tobacco control programs can identify the network's structure and connectedness of the agencies within the program. Once linked with program outcomes in future research, this analysis could be used to inform necessary development or organizational changes to tobacco control programs and produce better outcomes.

## Figures and Tables

**Table 1:** Characteristics of Five State Tobacco Control Networks, United States, 2002–2003

**State (Fiscal Year)**	**Network size and location**		**Funding status**		

	**Number of Partner Agencies**	**Number of Cities Where Partner Agencies Are Located**	**Total Tobacco Control Funds (millions)^a^**	**% Meeting CDC’s Minimum Recommendations^b^**	**RWJF Smokeless States Grant Received by Statewide Coalition**
Washington (2002)	14	7	$20.8	62	No grant received
Indiana (2003)	15	2	$33.9	97	No grant received
Wyoming (2003)	12	4	$4.2	57	$250,000
New York (2003)	15	11	$52.3	55	$450,000
Michigan (2003)	14	7	$5.3	10	$400,000

a Robert Wood Johnson Foundation Smokeless States grant funds are included in total tobacco control funds.

b Centers for Disease Control and Prevention (CDC) recommendations are outlined in *Best Practices for Comprehensive Tobacco Control Programs — August 1999* (1).

**Table 2:** Centrality Scores Used to Measure Communication Control in Five State Tobacco Control Networks, United States, 2002–2003^a^

**Washington**	

**Agency (type)**	**Score**
Washington State Department of Health Tobacco Prevention & Control Program (L)	26.0
American Lung Association - Washington State Branch (V)	14.6
American Cancer Society - Northwest Division (V)	11.8
Washington Alliance for Tobacco Control and Children's Health (SC)	7.2
King County Tobacco Control Coalition (C)	3.7
Washington Office of the Attorney General (O)	1.8
MWW/Savitt (O)	1.4
Sedgwick Rd (CT)	1.4
Group Health Cooperative, Center for Health Promotion (CT)	1.3
Puget Sound Educational Service District (CT)	1.2
Tobacco Free Spokane (C)	0.0
Tacoma-Pierce County Health Department (CT)	0.0
Puyallup Tribe (CT)	0.0
Washington State Hospital Association (O)	0.0
Mean (SD)	5.0 (7.3)
Density	0.44
Centralization index	22.6%

**Indiana**	

**Agency (type)**	**Score**
Indiana Tobacco Prevention and Cessation Agency (L)	25.1
Indiana State Department of Health (O)	8.5
American Cancer Society (V)	5.7
MZD Advertising (CT)	5.7
Tobacco Smart Indiana (SC)	3.6
Indiana Latino Institute, Inc (CT)	3.5
Smokefree Indiana (O)	2.0
Marion County Tobacco Control Program (C)	1.9
Indiana Minority Health Coalition (CT)	0.7
Indiana Tobacco Prevention and Cessation Agency Executive Board (O)	0.7
Indiana Black Expo (CT)	0.4
Smokefree Allen County (C)	0.2
American Heart Association (V)	0.1
Indiana State Medical Association (O)	0.1
Indiana Alliance of Boys & Girls Clubs (CT)	0.0
Mean (SD)	3.9 (6.2)
Density	0.50
Centralization index	22.7%

**Wyoming**	

**Agency (type)**	**Score**
Wyoming Department of Health Substance Abuse Division, Tobacco Prevention and Control Program (L)	45.6
Natrona County Tobacco Use Prevention Task Force (C)	10.0
Wyoming Tobacco Use Prevention (SC)	4.9
Making Laramie a Smoke Free Indoor Environment (C)	2.4
American Heart Association (V)	2.2
Wyoming Medical Society (O)	1.8
American Cancer Society (V)	1.1
Wyoming State Legislature (O)	1.1
Department of Education (O)	0.0
Partnership for Smoke-Free Families (CT)	0.0
Wyoming Statistical Analysis Center (CT)	0.0
Department of Maternal & Child Health (O)	0.0
Mean (SD)	5.7 (12.3)
Density	0.45
Centralization index	43.5%

**New York**	

**Agency (type)**	**Score**
New York State Department of Health Tobacco Control Program (L)	43.1
American Cancer Society (V)	16.7
American Lung Association (V)	5.8
American Heart Association (V)	3.7
Onondaga Cortland Madison BOCES (CT)	2.6
Coalition for a Tobacco-Free New York (SC)	1.7
Coalition for a Smoke-Free City (C)	0.5
Tobacco Action Coalition of Long Island (C)	0.5
New York Public Interest Research Group (V)	0.5
Tobacco Control Program Advisory Board (O)	0.5
Statewide Center for Healthy Schools (O)	0.5
Roswell Park Cancer Institute (CT)	0.0
Centers for Disease Control and Prevention, Office on Smoking and Health (O)	0.0
Bureau of Sanitation and Food Protection, Division of Environmental Health Protection, Center for Environmental Health (O)	0.0
Desmond Media (CT)	0.0
Mean (SD)	5.1 (11.0)
Density	0.39
Centralization index	40.8%

**Michigan**	

**Agency (type)**	**Score**
Michigan Department of Community Health Tobacco Section (L)	13.1
Tobacco Free Michigan Action Coalition (SC)	13.1
Center for Social Gerontology (CT)	13.1
American Lung Association (V)	4.1
Tobacco Control Law & Policy Consulting (O)	3.4
American Cancer Society (V)	1.0
Cristo Rey Community Center (V)	0.6
American Heart Association (V)	0.2
Marquette County Tobacco-Free Coalition (C)	0.0
Wayne County Smoking and Tobacco Intervention Coalition (C)	0.0
Faith Access to Community Economic Development Corporation (CT)	0.0
Genesee County Smokefree Multi-Agency Resource Team (C)	0.0
Center for Tobacco Use Prevention and Research (O)	0.0
University of Michigan Health System (O)	0.0
Mean (SD)	3.5 (5.2)
Density	0.58
Centralization index	10.4%

a *Centrality* provides a measure of how central an actor is in a network and is measured here using normalized betweenness; the higher the score, the greater control over communication flow in the network(30). An equation for calculating normalized betweenness is provided in Appendix B. L = lead agency, C = coalitions, SC= statewide coalition, CT = contractor, V = voluntary agency/advocacy group, O = other agency.

**Table 3:** Financial Influence Measured in Five State Tobacco Control Money Flow Networks, United States, 2002–2003^a^

**Washington**	

**Agency (type)**	**Score**
Washington State Department of Health Tobacco Prevention & Control Program (L)	0.74
Washington State Hospital Association (O)	0.05
American Cancer Society - Northwest Division (V)	0.00
American Lung Association - Washington State Branch (V)	0.00
Tacoma-Pierce County Health Department (CT)	0.00
Group Health Cooperative, Center for Health Promotion (CT)	0.00
Washington Office of the Attorney General (O)	0.00
Tobacco Free Spokane (C)	-0.05
Puget Sound Educational Service District (CT)	-0.05
Sedgwick Rd (CT)	-0.05
MWW/Savitt (O)	-0.05
King County Tobacco Control Coalition (C)	-0.16
Puyallup Tribe (CT)	-0.16
Washington Alliance for Tobacco Control and Children's Health (SC)	-0.26
Standard deviation	0.229
Density	0.08

**Indiana**	

**Agency (type)**	**Score**
Indiana Tobacco Prevention and Cessation Agency (L)	0.48
Indiana State Department of Health (O)	0.17
Smokefree Allen County (C)	0.13
American Heart Association (V)	0.00
Tobacco Smart Indiana (SC)	0.00
Indiana Tobacco Prevention and Cessation Agency Executive Board (O)	0.00
Indiana State Medical Association (O)	-0.03
MZD Advertising (CT)	-0.03
American Cancer Society (V)	-0.04
Marion County Tobacco Control Program (C)	-0.07
Indiana Alliance of Boys & Girls Clubs (CT)	-0.07
Smokefree Indiana (O)	-0.07
Indiana Latino Institute, Inc (CT)	-0.11
Indiana Black Expo (CT)	-0.12
Indiana Minority Health Coalition (CT)	-0.23
Standard deviation	0.163
Density	0.10

**Wyoming**	

**Agency (type)**	**Score**
Wyoming State Legislature (O)	0.46
Wyoming Department of Health Substance Abuse Division, Tobacco Prevention and Control Program (L)	0.23
American Cancer Society (V)	0.08
American Heart Association (V)	0.08
Wyoming Medical Society (O)	0.08
Department of Education (O)	0.00
Department of Maternal & Child Health (O)	-0.08
Making Laramie a Smoke Free Indoor Environment (C)	-0.15
Natrona County Tobacco Use Prevention Task Force (C)	-0.15
Wyoming Statistical Analysis Center (CT)	-0.15
Partnership for Smoke-Free Families (CT)	-0.15
Wyoming Tobacco Use Prevention (SC)	-0.23
Standard deviation	0.198
Density	0.07

**New York**	

**Agency (type)**	**Score**
New York State Department of Health Tobacco Control Program (L)	0.23
Centers for Disease Control and Prevention, Office on Smoking and Health (O)	0.16
Tobacco Action Coalition of Long Island (C)	0.11
American Heart Association (V)	0.01
Bureau of Sanitation and Food Protection, Division of Environmental Health Protection, Center for Environmental Health (O)	0.00
Tobacco Control Program Advisory Board (O)	0.00
Desmond Media (CT)	-0.01
Onondaga Cortland Madison BOCES (CT)	-0.01
Coalition for a Smoke-Free City (C)	-0.02
Statewide Center for Healthy Schools (O)	-0.03
Coalition for a Tobacco-Free New York (SC)	-0.06
American Cancer Society (V)	-0.07
Roswell Park Cancer Institute (CT)	-0.08
New York Public Interest Research Group (V)	-0.11
American Lung Association (V)	-0.12
Standard deviation	0.098
Density	0.10

**Michigan**	

**Agency (type)**	**Score**
Michigan Department of Community Health Tobacco Section (L)	0.52
Center for Social Gerontology (CT)	0.07
Center for Tobacco Use Prevention and Research (O)	0.07
American Cancer Society (V)	0.03
American Heart Association (V)	0.03
American Lung Association (V)	0.03
Marquette County Tobacco-Free Coalition (C)	0.00
Wayne County Smoking and Tobacco Intervention Coalition (C)	0.00
Cristo Rey Community Center (CT)	0.00
Faith Access to Community Economic Development Corporation (CT)	0.00
Genesee County Smokefree Multi-Agency Resource Team (C)	0.00
University of Michigan Health System (O)	-0.03
Tobacco Control Law & Policy Consulting (O)	-0.07
Tobacco Free Michigan Action Coalition (SC)	-0.66
Standard deviation	0.236
Density	0.10

a Taylor’s influence provides a measure of the amount of *financial influence* one agency has over others; a negative value indicates a preponderance of receiving money over sending, a positive value indicates a preponderance of sending over receiving, and a neutral value indicates a balance of sending and receiving; the greater the positive value, the greater the financial influence(31). An equation for calculating Taylor’s influence is provided in Appendix B. L = lead agency, C = coalitions, SC= statewide coalition, CT = contractor, V = voluntary agency/advocacy group, O = other agency.

**Table 4:** Prestige as a Measure of Relationship Productivity in Five State Tobacco Control Networks, United States, 2002-2003^a^

**Washington**	

**Agency (type)**	**Score**
Washington State Department of Health Tobacco Prevention & Control Program (L)	69.2
American Lung Association - Washington State Branch (V)	46.2
Washington Office of the Attorney General (O)	46.2
Group Health Cooperative, Center for Health Promotion (CT)	38.5
MWW/Savitt (O)	30.8
Washington Alliance for Tobacco Control and Children's Health (SC)	30.8
Tobacco Free Spokane (C)	23.1
Tacoma-Pierce County Health Department (CT)	23.1
American Cancer Society - Northwest Division (V)	15.4
Puget Sound Educational Service District (CT)	15.4
Sedgwick Rd (CT)	15.4
Washington State Hospital Association (O)	15.4
King County Tobacco Control Coalition (C)	7.7
Puyallup Tribe (CT)	0.0
Density	0.27

**Indiana**	

**Agency (type)**	**Score**
Indiana Tobacco Prevention and Cessation Agency (L)	85.7
Indiana State Department of Health (O)	57.1
American Cancer Society (V)	57.1
Indiana Tobacco Prevention and Cessation Agency Executive Board (O)	57.1
Smokefree Indiana (O)	42.9
Indiana State Medical Association (O)	42.9
MZD Advertising (CT)	42.9
American Heart Association (V)	35.7
Tobacco Smart Indiana (C)	28.6
Smokefree Allen County (C)	28.6
Indiana Minority Health Coalition (CT)	28.6
Indiana Latino Institute, Inc (CT)	28.6
Marion County Tobacco Control Program (C)	21.4
Indiana Black Expo (CT)	21.4
Indiana Alliance of Boys & Girls Clubs (CT)	0.0
Density	0.39

**Wyoming**	

**Agency (type)**	**Score**
Wyoming Tobacco Use Prevention (SC)	81.8
American Heart Association (V)	54.5
Wyoming Statistical Analysis Center (CT)	54.5
Wyoming Department of Health Substance Abuse Division, Tobacco Prevention and Control Program (L)	45.5
Natrona County Tobacco Use Prevention Task Force (C)	45.5
Partnership for Smoke-Free Families (CT)	45.5
American Cancer Society (V)	36.4
Making Laramie a Smoke Free Indoor Environment (C)	36.4
Department of Education (O)	27.3
Wyoming Medical Society (O)	18.2
Department of Maternal & Child Health (O)	18.2
Wyoming State Legislature (O)	9.1
Density	0.39

**New York**	

**Agency (type)**	**Score**
American Cancer Society (V)	57.1
American Lung Association (V)	57.1
New York State Department of Health Tobacco Control Program (L)	50.0
American Heart Association (V)	42.9
Coalition for a Tobacco-Free New York (SC)	35.7
Coalition for a Smoke-Free City (C)	35.7
Tobacco Action Coalition of Long Island (C)	28.6
New York Public Interest Research Group (V)	21.4
Centers for Disease Control and Prevention, Office on Smoking and Health (O)	14.3
Statewide Center for Healthy Schools (O)	14.3
Bureau of Sanitation and Food Protection, Division of Environmental Health Protection, Center for Environmental Health (O)	7.1
Tobacco Control Program Advisory Board (O)	7.1
Onondaga Cortland Madison BOCES (CT)	7.1
Roswell Park Cancer Institute (CT)	0.0
Desmond Media (CT)	0.0
Density	0.25

**Michigan**	

**Agency (type)**	**Score**
Tobacco Free Michigan Action Coalition (SC)	92.3
Michigan Department of Community Health Tobacco Section (L)	76.9
American Lung Association (V)	76.9
Center for Social Gerontology (CT)	53.8
American Heart Association (V)	38.5
Genesee County Smokefree Multi-Agency Resource Team (C)	38.5
American Cancer Society (V)	30.8
Marquette County Tobacco-Free Coalition (C)	30.8
Cristo Rey Community Center (CT)	30.8
Faith Access to Community Economic Development Corporation (CT)	30.8
Tobacco Control Law & Policy Consulting (O)	30.8
Wayne County Smoking and Tobacco Intervention Coalition (C)	23.1
Center for Tobacco Use Prevention and Research (O)	15.4
University of Michigan Health System (O)	7.7
Density	0.41

a *Prestige* provides a measure of relationship productivity and was measured using normalized indegree; the higher the indegree score, the more highly productive the relationship was considered to be and the higher the prestige (28). An equation for calculating normalized indegree is provided in Appendix B. L = lead agency, C = coalitions, SC= statewide coalition, CT = contractor, V = voluntary agency/advocacy group, O = other agency.

**Table 5:** Correlation of Contact and Productivity Network Variables, Tobacco State Control Networks, United States, 2002–2003

	**Contact/Productivity**	

***State***	***Pearson Correlation***	***P***
Washington	.330	.002
Indiana	.568	<.001
Wyoming	.497	<.001
New York	.773	<.001
Michigan	.641	<.001

## Appendix A

**Example of Network Data Collection Form Used in Interviews, State Tobacco Control Networks, United States, 2002–2003**

**Table:**

	**1. How often does your agency have contact (such as meetings, phone calls, or e-mails) with [AGENCY]?**						
	
**Agency**	**No Contact**	**Daily**	**Weekly**	**Monthly**	**Quarterly**	**Yearly**	**Unable to Answer**
**State Department of Health**	1	2	3	4	5	6	8
**American Cancer Society**	1	2	3	4	5	6	8
**American Heart Association**	1	2	3	4	5	6	8
**WY Tobacco Use Prevention**	1	2	3	4	5	6	8
**Making Laramie A Smoke Free Indoor Environment**	1	2	3	4	5	6	8
**Natrona County Tobacco Use Prevention Task Force**	1	2	3	4	5	6	8
**WY Medical Society**	1	2	3	4	5	6	8
**WY Statistical Analysis Center**	1	2	3	4	5	6	8
**Partnership for Smoke Free Families**	1	2	3	4	5	6	8
**Department of Maternal & Child Health**	1	2	3	4	5	6	8
**Department of Education**	1	2	3	4	5	6	8
**WY State Legislature**	1	2	3	4	5	6	8

	**2. How productive do you feel the relationship is between your agency and [AGENCY]?**						
	
**Agency**	**Counter-productive**	**Very Unproductive**	**Somewhat Unproductive**	**Neutral**	**Somewhat Productive**	**Very Productive**	**UTA**
**State Department of Health**	0	1	2	3	4	5	8
**American Cancer Society**	0	1	2	3	4	5	8
**American Heart Association**	0	1	2	3	4	5	8
**WY Tobacco Use Prevention**	0	1	2	3	4	5	8
**Making Laramie A Smoke Free Indoor Environment**	0	1	2	3	4	5	8
**Natrona County Tobacco Use Prevention Task Force**	0	1	2	3	4	5	8
**WY Medical Society**	0	1	2	3	4	5	8
**WY Statistical Analysis Center**	0	1	2	3	4	5	8
**Partnership for Smoke Free Families**	0	1	2	3	4	5	8
**Department of Maternal & Child Health**	0	1	2	3	4	5	8
**Department of Education**	0	1	2	3	4	5	8
**WY State Legislature**	0	1	2	3	4	5	8

	**3. Does your agency primarily send or receive money* with [AGENCY]? *[includes any formal funds (e.g., grants, membership dues)]**				
	
**Agency**	**Send**	**Receive**	**Both (send & receive)**	**None of the above**	**UTA**
**State Department of Health**	1	2	3	4	8
**American Cancer Society**	1	2	3	4	8
**American Heart Association**	1	2	3	4	8
**WY Tobacco Use Prevention**	1	2	3	4	8
**Making Laramie A Smoke Free Indoor Environment**	1	2	3	4	8
**Natrona County Tobacco Use Prevention Task Force**	1	2	3	4	8
**WY Medical Society**	1	2	3	4	8
**WY Statistical Analysis Center**	1	2	3	4	8
**Partnership for Smoke Free Families**	1	2	3	4	8
**Department of Maternal & Child Health**	1	2	3	4	8
**Department of Education**	1	2	3	4	8
**WY State Legislature**	1	2	3	4	8

## Appendix B

**Calculations for Normalized Betweenness, Taylor’s Influence, and Normalized Indegree** *Normalized betweenness* (C’_B_) is calculated using the following equation:

where g = the number of agencies in the network, g_jk_(n_i_) = the number of geodesics between j and k containing agency i, and g_jk_ = the total number of geodesics between j and k. A geodesic is the shortest path between two agencies. The denominator represents the number of pairs of actors not including n_i_ (30).

*Taylor’s influence* counts the total direct connections between actors and applies an attenuation factor based on the length of each connection. The balance between each actor sending connections (row marginals) and receiving connections (column marginals) is then calculated (31).

*Normalized indegree* (P’_D_[n_i_]) is calculated using the following equation:

where the numerator represents the number of choices received by n_i_ (the number of arrows pointing toward n_i_). The denominator represents the number of possible choices that could be received by n_i_ (28).

## Appendix C

**Agency Names and Abbreviations, State Tobacco Control Networks**

**Table:**

**Agency name**	**Abbreviation**

**Washington**	

Washington State Department of Health Tobacco Prevention & Control Program	WA DOH
American Cancer Society – Northwest Division	ACS
American Lung Association – Washington State Branch	ALA
King County Tobacco Control Coalition	King CC
Tobacco Free Spokane	TF Spokane
Tacoma-Pierce County Health Department	Tacoma-Pierce HD
Puget Sound Educational Service District	Puget ESD
Puyallup Tribe	Puyallup
Group Health Cooperative, Center for Health Promotion	GHP-CHP
Sedgwick Rd	Sedgwick
MWW/Savitt	MWW
Washington Alliance for Tobacco Control and Children’s Health	WATCH
Washington Office of the Attorney General	WA AG
Washington State Hospital Association	WA SHA

**Indiana**	

Indiana Tobacco Prevention and Cessation Agency	ITPC
Indiana Tobacco Prevention and Cessation Agency Executive Board	ITPC Board
American Cancer Society	ACS
American Heart Association	AHA
Indiana Alliance of Boys & Girls Clubs	B&G Clubs
Indiana Black Expo	Black Expo
Indiana Latino Institute, Inc	Latino Inst
Indiana Minority Health Coalition	IMHC
Indiana State Department of Health	DOH
Indiana State Medical Association	ISMA
MZD Advertising	MZD
Marion County Tobacco Control Program	MC TCP
Smokefree Allen County	SFAC
Smokefree Indiana	SF IN
Tobacco Smart Indiana	TS IN

**Wyoming**	

Wyoming Department of Health Substance Abuse Division, Tobacco Prevention and Control Program	DOH TPCP
American Cancer Society	ACS
American Heart Association	AHA
Wyoming Tobacco Use Prevention	WY TUP
Making Laramie a Smoke Free Indoor Environment	Laramie
Natrona County Tobacco Use Prevention Task Force	Natrona
Wyoming Medical Society	WY MS
Wyoming Statistical Analysis Center	WY SAC
Partnership for Smoke-Free Families	PSFF
Department of Maternal & Child Health	DMCH
Department of Education	DOE
Wyoming State Legislature	Legislature

**New York**	

New York State Department of Health Tobacco Control Program	DOH TCP
American Cancer Society	ACS
American Heart Association	AHA
American Lung Association	ALA
Coalition for a Tobacco-Free New York	Coalition TF NY
Coalition for a Smoke-Free City	Coalition SF City
Tobacco Action Coalition of Long Island	Coalition LI
Centers for Disease Control and Prevention, Office on Smoking and Health	CDC-OSH
Roswell Park Cancer Institute	Roswell
Desmond Media	Desmond
Onondaga Cortland Madison BOCES	OCM BOCES
New York Public Interest Research Group	NYPIRG
Bureau of Sanitation and Food Protection, Division of Environmental Health Protection, Center for Environmental Health	Env Health
Statewide Center for Healthy Schools	Healthy Schools
Tobacco Control Program Advisory Board	Board

**Michigan**	

Michigan Department of Community Health Tobacco Section	MDCH TS
American Cancer Society	ACS
American Heart Association	AHA
American Lung Association	ALA
Center for Social Gerontology	Gerontology
Center for Tobacco Use Prevention and Research	CTUPR
Cristo Rey Community Center	Cristo
Faith Access to Community Economic Development Corporation	FACED
Genesee County Smokefree Multi-Agency Resource Team	Genesee
Marquette County Tobacco-Free Coalition	Marquette
Tobacco Control Law & Policy Consulting	Law & Policy
Tobacco Free Michigan Action Coalition	TFMAC
University of Michigan Health System	U of M Health
Wayne County Smoking and Tobacco Intervention Coalition	Wayne
